# Laboratorial training of examiners for using a visual caries detection system in epidemiological surveys

**DOI:** 10.1186/1472-6831-13-49

**Published:** 2013-10-03

**Authors:** Chaiana Piovesan, Bruna LP Moro, Juan S Lara, Thiago M Ardenghi, Renata S Guedes, Ana E Haddad, Mariana M Braga, Fausto M Mendes

**Affiliations:** 1 Department of Pediatric Dentistry, School of Dentistry, University of São Paulo, Av. Lineu Prestes, 2227, São Paulo, SP 05508-000, Brazil; 2 Department of Stomatology, Universidade Federal de Santa Maria, Santa Maria, Rio Grande do Sul, Brazil

**Keywords:** ICDAS, Diagnosis, Dental caries, Epidemiologic surveys, Examiners, Calibration

## Abstract

**Background:**

In epidemiological surveys, a good reliability among the examiners regarding the caries detection method is essential. However, training and calibrating those examiners is an arduous task because it involves several patients who are examined many times. To facilitate this step, we aimed to propose a laboratory methodology to simulate the examinations performed to detect caries lesions using the International Caries Detection and Assessment System (ICDAS) in epidemiological surveys.

**Methods:**

A benchmark examiner conducted all training sessions. A total of 67 exfoliated primary teeth, varying from sound to extensive cavitated, were set in seven arch models to simulate complete mouths in primary dentition. Sixteen examiners (graduate students) evaluated all surfaces of the teeth under illumination using buccal mirrors and ball-ended probe in two occasions, using only coronal primary caries scores of the ICDAS. As reference standard, two different examiners assessed the proximal surfaces by direct visual inspection, classifying them in sound, with non-cavitated or with cavitated lesions. After, teeth were sectioned in the bucco-lingual direction, and the examiners assessed the sections in stereomicroscope, classifying the occlusal and smooth surfaces according to lesion depth. Inter-examiner reproducibility was evaluated using weighted kappa. Sensitivities and specificities were calculated at two thresholds: all lesions and advanced lesions (cavitated lesions in proximal surfaces and lesions reaching the dentine in occlusal and smooth surfaces).

**Results:**

Regarding the reproducibility, the mean (range) of kappa values was 0.781 (0.529–0.927) for occlusal surfaces, 0.568 (0.191–0.881) for smooth surfaces, and 0.844 (0.698–0.971) for proximal surfaces. Considering all lesions, sensitivity and specificity mean values were respectively 0.724 and 0.844 for occlusal, 0.635 and 0.943 for smooth and 0.658 and 0.927 for proximal surfaces. For detecting advanced lesions, sensitivities and specificities were 0.563 and 0.920 for occlusal, 0.670 and 0.985 for smooth, and 0.838 and 0.985 for proximal surfaces.

**Conclusion:**

The methodology purposed for training and calibration of several examiners designated for epidemiological surveys of dental caries in preschool children using the ICDAS is feasible, permitting the assessment of reliability and accuracy of the examiners previously to the survey´s development.

## Background

Methods for caries detection including assessment of cavitated and non-cavitated lesions have been recently used in oral health epidemiological surveys [1-5]. In this context, the International Caries Detection and Assessment System (ICDAS) is one of the visual scoring systems developed to assess the caries lesions at both thresholds [6,7]. Studies that used the ICDAS have showed its feasibility in laboratory and clinical studies [1,4,8-10]. Furthermore, the method has been employed in some epidemiological surveys to assess dental caries in primary teeth [1,2,4]. Nevertheless, considering that epidemiological surveys often involve several examiners, the inter-observer variability in the examinations is a matter of concern.

To improve the reliability of the data obtained by different examiners, training and calibration exercises are organized previously to the oral health epidemiological surveys. In these earlier sessions, some subjects are usually recruited and examined several times by all examiners in order to reach a satisfactory calibration among the dentists. The evaluation of calibration is based on data obtained from the assessment of a benchmark examiner, who is considered as ‘gold standard’ [3]. Therefore, the benchmark examiner is assumed to be error free; however, this person is actually no more than an experienced examiner. Taking into account that the ‘gold standard’ is an instrument or technique which reflects the ‘truth’, data obtained from calibration exercises with benchmark examiner should be used only to assess the level of agreement among examiners, and not the accuracy of the examiners [11].

Therefore, the evaluation of the performance of the examiners in epidemiological surveys has been generally checked through their reliability [1,2,4]. Considering that histological examination [12] and direct visual inspection of proximal surfaces [8] might be regarded as providing reference standard methods and would allow to calculate the accuracy of the examiners [11], we aimed to propose a methodology to simulate the examinations performed to detect caries lesions using the ICDAS in epidemiological surveys. We evaluated the feasibility of the methodology in training and calibration of the examiners, assessing their performances in terms of accuracy and reliability.

## Methods

Both Committees for Ethics in Research of Federal University of Santa Maria and School of Dentistry, University of São Paulo, Brazil approved the study (process number 0270.0.243.000-09). The methodology described in this paper was used to train and calibrate examiners to collect data about dental caries at non-cavitated and cavitated thresholds of preschool children in an epidemiological survey performed during the National Children’s Vaccination Day in Santa Maria, Brazil in 2010.

Briefly, in this survey, we investigated the effect of assessing caries lesions activity on the magnitude of caries parameters in an epidemiological survey of preschool children. For the evaluations, we opted to use the ICDAS, which is a system that records dental caries at both cavitated and non-cavitated thresholds. In the epidemiological survey, the system was associated with adjunct criteria to assess of caries lesion activity status. The system describes the several grades of dental caries and range of score 0 (Sound surface) to 6 (Extensive distinct cavity with visible dentine). Full details of the diagnostic criteria and codes have been previously reported [4,6,7]. More information about the methodology and data obtained in this epidemiological survey was published elsewhere [13].

Prior to the survey, training and calibration sessions were performed using a different methodology at the laboratory setting. However, in the in vitro training sessions, the examiners were orientated to use only the ICDAS scores and they did not employ the activity assessment. This methodology is the main focus of the present study and it will be described hereinafter.

### Training and calibration methodology

A total of 16 examiners took part in the study. All examiners were graduate dental students and they had no previous experience in using the ICDAS. The responsible for all training sessions was a benchmark examiner (CP) who has been extensively trained and calibrated for using the ICDAS by a member of the ICDAS committee; moreover, she has participated in previous studies using the system.

First, the training sessions included theoretical explanations and discussion using clinical photographic examples. The images comprised visual examples of all components of the criteria.

**Figure 1:**
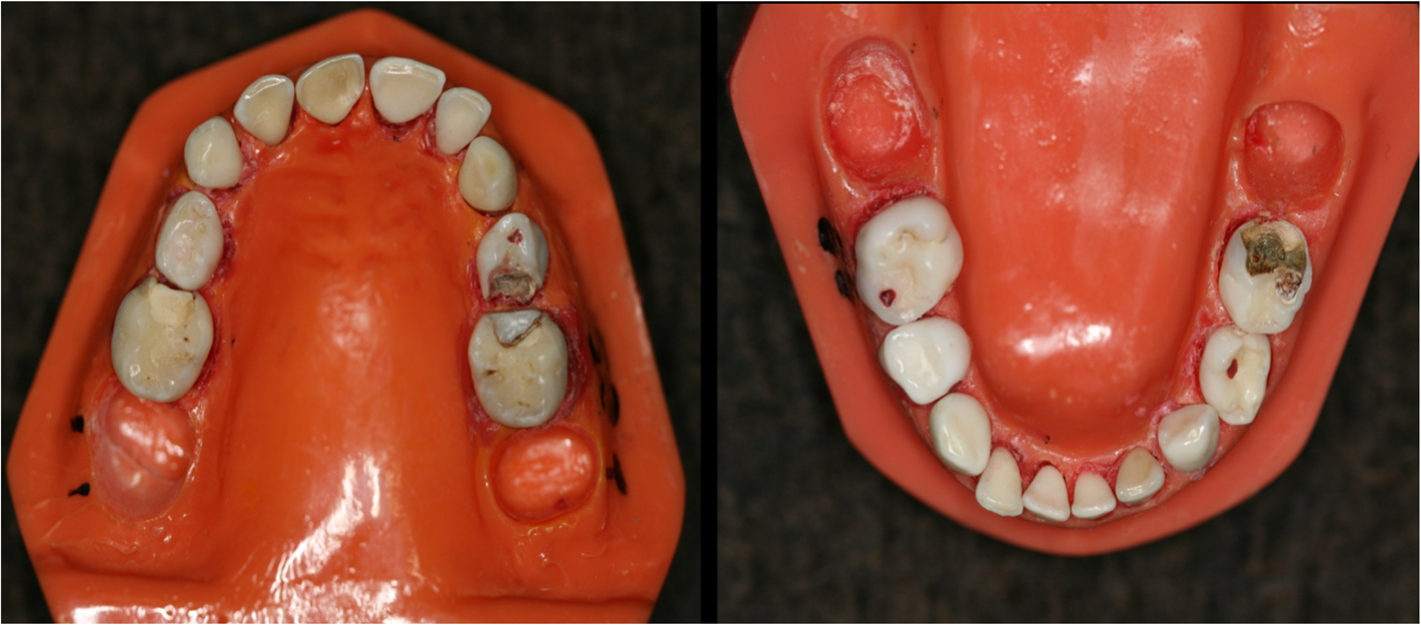
Dental arch models used to train and calibrate examiners designated to epidemiological surveys in detecting caries lesions using the International Caries Detection and Assessment System.

Subsequently, 67 exfoliated or extracted primary teeth were selected by the benchmark examiner. The chosen teeth presented a variety of instances of lesions related to ICDAS scores. All ICDAS codes (0 to 6) were represented in the sample. We also included some teeth with restored surfaces. Then, the teeth were set in seven arch models to simulate mouths in primary dentition (3 lower and 4 upper arches). Care was taken to simulate as best as possible the order and correct side of the teeth. The presence of contact points was confirmed using dental floss. Two mouths were set with the absence of three specimens to simulate missed teeth. To avoid dehydration of the samples, gauze pads soaked in distilled water was placed in closed containers with the models. A lower and an upper dental arch with the teeth used during the training and calibration sessions can be observed in the Figure 1.

After, the examiners evaluated the teeth in a dental unit using only the coronal primary caries scores of the ICDAS. The restored and missed teeth were also recorded using simplified codes (‘R’ for restored and ‘M’ for missed surface or teeth). The models were positioned about 30 cm from the examiners’ eyes. The assessments were performed using a mouth mirror and a ballpoint probe, with the aid of a light reflector. The teeth were first examined wet, and then they were dried for 5 seconds with compressed air. The examiners were orientated to not move the models.

Two sessions occurred using this methodology. The first session was performed to teach the examiners about the procedure, and the second session, a week later, was conducted to obtain the data about the performance.

### Validation procedures

After the training and calibration sessions, we performed the procedures to validate the clinical examinations. We validated all surfaces of anterior and posterior teeth. For this, the teeth were removed of arch models and each tooth was evaluated independently.

As reference standard methods, we used direct visual inspection for proximal surfaces and histological examination for occlusal, buccal and lingual surfaces. We opt to cut the teeth in the bucco-lingual direction in order to assess the histological sections of the occlusal and smooth surfaces. To validate the proximal surfaces, we used the direct visual inspection. Similar procedures were previously used in other studies [8,14].

The direct visual inspection of the proximal surfaces was performed after the removal of the tooth from the dental arch model [8]. Two different examiners (CP and BLPM) assessed the surfaces with no magnification and after air drying for 5 s, with aid of a ballpoint probe and light reflector. The examiners independently classified the surfaces as sound, with either non-cavitated caries lesions or with cavitated caries lesions. The examiners were not aware from the results previously obtained when teeth were examined positioned in the arch model. In case of disagreements, the examiners evaluated the surfaces again in a joint session until they reached a consensus.

For the buccal, lingual and occlusal surfaces, the reference standard method was the histological examination. For this, the samples were embedded in acrylic resin. After, teeth were sectioned in the bucco-lingual direction (approximately 800 μm thick) with a diamond disc mounted in a cutting machine (Isomet 5000, Buehler, Lake Bluff, IL, USA). Four to seven sections per tooth were made. All sections were assessed separately by two examiners using a stereomicroscope at 16–40 x magnification under reflecting light (Stereomicroscope M80; Leica Microsystems, Heidelberg, Germany). They classified the occlusal and smooth surfaces according to lesion depth using a 5-point scale: D0 = no caries; D1 = caries lesion limited to the outer half of the enamel; D2 = caries extending into the inner half of the enamel but not to the amelodentinal junction; D3 = caries limited to the outer half of the dentine; D4 = caries involving the inner half of the dentine [15]. The worst score for each surface was given. In cases where disagreements between examiners occurred, new examinations were performed and a consensus decision was reached.

### Statistical analysis

The statistical unit was the dental surface. All analyses were performed separately for proximal, occlusal and smooth surfaces. Inter-examiner reproducibility was firstly calculated through intraclass correlation coefficient (ICC) and respective 95% confidence intervals, considering all examiners together at the same analysis. Then, the inter-examiner reliability was calculated considering the ICDAS scores using quadratic weighted Kappa. We performed the kappa evaluations considering pairs of examiners, totaling 120 kappa values for each group of surfaces. ICC and Kappa values for all surfaces together were also obtained considering all surfaces at the same analyses.

In order to assess the sensitivity and specificity, data obtained from 16 examiners were compared to the results obtained with the reference standard methods: direct visual inspection for proximal surfaces and histological classification for occlusal and smooth surfaces. The mean, standard deviation and range of sensitivity and specificity were determined at the following diagnostic thresholds: D1 (all lesions) and D3 (more advanced caries lesions represented by cavitated lesions in proximal surfaces and lesions reaching the dentine in occlusal and smooth surfaces). At D1 threshold, lesions classified as score 0 of ICDAS were considered as sound and lesions classified as scores 1 to 6 were considered as decayed. When we considered more advanced caries lesions (D3 threshold), surfaces scored as 0 to 2 by the ICDAS were considered sound, while surfaces classified as scores 3 to 6 were considered as decayed. Missed and restored surfaces were not considered in the statistical analyses.

## Results

With regard to the reliability, the examiners achieved higher ICC values at both occlusal and proximal surfaces compared to that obtained at smooth surfaces. Weighted kappa values were higher at proximal surfaces. The examinations performed at occlusal surfaces presented higher figures than those obtained for buccal and lingual surfaces. Furthermore, the range of kappa values obtained at occlusal and proximal surfaces was narrower than that obtained at smooth surfaces. The overall ICC and weighted kappa values considering all surfaces together were higher than 0.7 (Table 1).

**Table 1:** Interexaminer reproducibility from examiners using the International Caries Detection and Assessment System in detecting caries lesions in primary teeth during training and calibration session for epidemiological survey

	**Intraclass correlation coefficient**	**Weighted kappa values**		
	**(95% confidence interval)**	**Mean**	**Standard deviation**	**Range**
**Occlusal surfaces**	0.828 (0.743 to 0.901)	0.781	0.078	0.529 – 0.927
**Buccal/lingual surfaces**	0.587 (0.526 to 0.651)	0.568	0.134	0.191 – 0.881
**Proximal surfaces**	0.833 (0.797 to 0.867)	0.844	0.058	0.698 – 0.971
**All surfaces**	0.790 (0.761 to 0.817)	0.731	0.152	0.191 – 0.971

With the validation procedure of the proximal surfaces (direct visul inspection), we found 92 sound surfaces (68.7%), 25 surfaces with white spot lesions (18.7%), 15 cavitated lesions (11.2%) and two restored proximal surfaces (1.5%). For the smooth surfaces, the histological validation showed that 121 were sound (90.3%), 5 surfaces presented initial enamel lesions (3.7%), one lesion was classified as D2 (0.7%), 3 (2.2%) and 4 (3.0%) surfaces had initial and advanced dentin caries lesions, respectively. For occlusal surfaces, 4 were considered as sound in the histological validation (14.3%), 8 surfaces presented initial enamel lesions (28.6%), 6 surfaces had advanced enamel lesions (21.4%), 5 (17.9%) and 4 (14.3%) presented initial and advanced dentin caries lesions, respectively, and 1 surface were restored (3.6%).

At both thresholds, the examiners usually obtained higher specificities than sensitivities for all surfaces. Considering all lesions, mean of sensitivity values was higher at occlusal surfaces. On the other hand, specificity was higher when the examinations were carried out at smooth surfaces. Considering more advanced caries lesions (D3 threshold), specificities obtained in proximal and smooth surfaces were higher than 0.94 for all examiners. The worse accuracy at this threshold was obtained at occlusal surfaces, since the mean of sensitivity and specificity obtained in this surface was lower than those obtained in the other surfaces (Table 2).

**Table 2:** Accuracy in terms of sensitivity and specificity of examiners using the International Caries Detection and Assessment System (ICDAS) in detecting caries lesions in primary teeth during training and calibration session for epidemiological survey

	**Mean**	**SD**	**Range**
All lesions*			
**Occlusal surfaces**			
Sensitivity	0.724	0.098	0.583 – 0.917
Specificity	0.844	0.239	0.250 – 1.000
**Buccal/lingual surfaces**			
Sensitivity	0.635	0.189	0.231 – 0.923
Specificity	0.943	0.042	0.818 – 0.992
**Proximal surfaces**			
Sensitivity	0.658	0.107	0.500 – 0.833
Specificity	0.927	0.051	0.837 – 0.978
More advanced lesions**			
**Occlusal surfaces**			
Sensitivity	0.563	0.072	0.500 – 0.700
Specificity	0.920	0.097	0.667 – 1.000
**Buccal/lingual surfaces**			
Sensitivity	0.670	0.249	0.286 – 1.000
Specificity	0.985	0.015	0.945 – 1.000
**Proximal surfaces**			
Sensitivity	0.838	0.063	0.706 – 0.941
Specificity	0.985	0.011	0.949 – 0.991

SD = Standard deviation;

*score 0 of ICDAS = sound; scores 1 to 6 of ICDAS = decayed;

**scores 0 to 2 of ICDAS = sound; scores 3 to 6 of ICDAS = decayed.

## Discussion

This study has proposed a methodology which can be used to train and calibrate several examiners designated to dental caries epidemiological surveys in preschool children. Although previous studies had assessed the diagnostic accuracy in dental survey using the in vitro validity of a different diagnostic system [14,16], this is the first study that proposed a laboratory methodology to simulate the examinations performed to detect caries lesions using the ICDAS in epidemiological surveys with preschool children.

Generally, in studies that involve multiple examiners, a benchmark examiner is used as *gold standard* in calibration exercises [3,17]. For calibration purposes, the level of agreement between scores obtained by the examiners and the benchmark one (interexaminer agreement) or taking into account repeated examinations by the same examiner (intraexaminer agreement) is obtained by assessment of a small group of subjects. Although this methodology has been widely used, the calibration using group of subjects is more difficulty when the survey involves preschool children.

To illustrate this difficulty, in our study each child would be evaluated by all 16 examiners at the same day. Moreover, to evaluate the intraexaminer agreement, the same children would be evaluated 16 times again after a week. Despite the difficulty, we carried out this type of calibration after the in vitro assessments using 10 children. We opt to do this additional in vivo step because of the epidemiological survey, since our in vitro methodology has not been validated yet.

The kappa values obtained in the clinical sessions were higher than 0.86 and 0.77 for the inter and intraexaminer agreement, respectively [13]. In a previous survey organized by our group using similar procedures of training and calibration, the overall weighted kappa value obtained by 6 examiners compared to assessments performed by a benchmark examiner was 0.78 [4].

The proposed methodology could be an alternative to shorten or eliminate the necessity of using children for calibrating examiners designated for dental caries surveys, mainly when several examiners are required. However, we could not evaluate if this training improved the accuracy and reliability of the examiners in detecting caries lesions during the survey. Although we have obtained good reliability values in our studies [4,13], further research is necessary to evaluate if this laboratory training methodology is responsible for these performances.

Moreover, as the ICDAS presents several grades of dental caries diagnostic, is difficult to find all scores in the clinical setting using few children. Then, with our methodology, it is possible to choose teeth presented a variety of instances of lesions related to ICDAS scores to training the examiners. Another advantage is that, in multicenter studies, is possible to transport the arch models for different places guaranteeing standardized training procedures with different examiners of the several centers included in the study. This method could decrease the discrepancies among examiners and among the centers.

Our study simulating an epidemiological survey showed that the examiners using the ICDAS exhibit good validity and acceptable reliability in detecting caries lesions in primary teeth. Nevertheless, one could affirm that the detection of caries lesions in a laboratory setting would be easier since bacterial plaque, acquired pellicle saliva and soft tissues are absent. Furthermore, clinical examinations in preschool children can present other difficult, such as child’s behavior and limited mouth opening. Although this assertion is true, a previous study showed similarity in the accuracy of visual inspection performed under clinical and laboratory settings [18]. Unfortunately, there are no reports about the criterion validity of the ICDAS performed by several examiners in similar conditions of our study. Therefore, our findings can only be compared with laboratory and clinical studies simulating the dental office setting.

The examiners of our study achieved high interexaminer kappa and ICC values in occlusal and proximal surfaces using ICDAS criteria. These figures were similar than those obtained in previous in vitro and in vivo studies [8,9,19-21]. However, for smooth surface, the same pattern was not observed. These results were surprisingly, since it would be expected that the direct visual inspection of the smooth surfaces would be easier than in occlusal and proximal surfaces. Probably, the low values of interexaminer reproducibility in smooth surfaces are due to the difficulty of the examiners in distinguishing sound and surfaces with initial caries lesions. No previous study has investigated the performance of the ICDAS in detecting smooth-surface caries lesions in primary teeth. In occlusal surfaces, some sound sites have been incorrectly scored as lesions presenting clinical characteristics of incipient caries [9], but studies should be carried out to investigate this possibility in smooth surfaces.

Concerning the accuracy, the validity of caries lesions detection at D1 threshold did not seem to be affected by the utilization of several examiners, since the values were similar to previous investigations [8,20-24].

At more advanced lesions threshold, the examiners using the ICDAS showed good and similar performance for all surfaces. In general, visual inspection presents high specificity values [9,12,19,23], corroborating our results. On the other hand, the sensitivities were low in occlusal and smooth surfaces. This trend was mainly observed in the surfaces that were validated by histological examination. The low values of sensitivity indicate a high number of false negative results. This finding was probably due to the reference standard method. The histological examination is able to detect small mineral changes in dentine, even in non-cavitated lesions. This alteration, however, could not be noticeable by the clinical examination, leading to a false-negative result and higher values of sensitivity.

In fact, this low sensitivity, and consequently, high specificity, is an advantage. For dental caries, methods with high specificity values are preferable because the higher occurrence of false-positive results could lead to unnecessary operative treatment. At the public health context, a high number of false-positives would result in increased costs to the government with unnecessary operative treatments.

The agreement between examiners and reference examiners has been usually measured in previous studies by different techniques, such as kappa statistics, percentage agreement, sensitivity and specificity. These methods present different properties and specific shortcomings [11]. Our methodology permits to evaluate the performance of the examiners in terms of both reliability, considering the agreement between the examiners, and validity, taking into account the results of the reference standard methods. The performance could be used to select the examiners with best accuracy to participate of the data collection in the main study. In our study, one examiner obtained very low performance in assessing smooth surfaces; thus, this examiner did not participate in the epidemiological survey [13].

Other possibility would be to correct the dental caries parameters obtained in the survey for possible examiner’s misclassifications. This type of correction was already proposed considering the agreement obtained by the examiners [25]. The feasibility and validity of this adjustment based on our methodology, however, must be tested in further occasions.

## Conclusion

In conclusion, the methodology purposed for training and calibration of several examiners designated for epidemiological surveys of dental caries in preschool children using the ICDAS is feasible.

## Competing interests

The authors certify that they have no financial or other personal interest in any product, service or company mentioned in this article.

## Authors’ contributions

CP was the benchmark examiner, was responsible to validate the sample and wrote the manuscript. BLPM has been involved in the in vitro experiments. JSL has also been involved in the in vitro experiments and wrote the manuscript. TMA and RSG performed the statistical analysis and revised the manuscript. AEH and MMB have been involved in the interpretation of the results and revised the manuscript. FMM had the original idea, coordinated the study and revised the manuscript. All authors read and approved the final manuscript.

## Pre-publication history

The pre-publication history for this paper can be accessed here:

http://www.biomedcentral.com/1472-6831/13/49/prepub
